# Bone Marrow Infiltration in Rosai–Dorfman Disease

**DOI:** 10.1155/2022/3420311

**Published:** 2022-12-15

**Authors:** Fatima Zahra Rahali, Fatima Taher, Houda Nassih, Sanae Sayagh

**Affiliations:** ^1^Hematology Laboratory, University Hospital Centre Mohammed VI, Marrakesh, Morocco; ^2^Faculty of Medicine and Pharmacy, Marrakesh, Morocco; ^3^Department of Pediatrics, University Hospital Centre Mohammed VI, Marrakesh, Morocco

## Abstract

Rosai–Dorfman disease (RDD) is a rare and benign nonLangerhans cell histiocytosis. RDD commonly affects children and young adults typically presenting with massive, painless, and bilateral cervical lymphadenopathy. Extranodal involvement is present in about 40% of cases, and bone marrow infiltration is rare and unusual. RDD prognosis is usually good, however, involvement of bone marrow is associated with poor prognosis. We report a case of RDD with bone marrow involvement occurring in a 5-year-old female. She was admitted for asthenia, gingival bleeding, and diffuse bone pain without fever. Physical examination showed pallor, petechiae over the abdomen, painless and bilateral cervical lymphadenopathy, and several bony nodules, without splenomegaly or hepatomegaly. Cell blood count revealed microcytic hypochromic anemia with thrombocytopenia. Bone marrow aspiration showed infiltration by large histiocytes with hypochromatic nuclei and abundant vacuolated cytoplasm without emperipolesis. Biopsies of bone marrow, lymph node, and bone revealed immunohistochemical features of RDD: the histiocytes were positive for CD68 and S100 protein, but negative for CD1a. The patient initially received symptomatic treatment. One week after admission, she died of septic shock before the final establishment of RDD diagnosis. This case report highlights that bone marrow involvement in RDD is rare and associated with poor prognosis. We also aim to emphasize the obligation of bone marrow exploration in patients with RDD and presenting cytopenias in order to make an early diagnosis of bone marrow infiltration.

## 1. Introduction

Rosai–Dorfman disease (RDD) is a rare and benign nonLangerhans cell histiocytosis [1–3]. The pathophysiology of this disorder is poorly understood [4]. According to the 2016 revised histiocytosis classification, RDD belongs to the “R Group” of histiocytosis which includes familial and sporadic RDD, while cutaneous RDD belongs to the “C Group” of histiocytosis [5]. RDD affects habitually children and young adults presenting with bilateral, painless cervical lymphadenopathy [4]. Extranodal involvement is present in about 40% of cases [4]. Bone marrow infiltration, as an extranodal manifestation, is rare and associated with poor prognosis [6, 7].

We report here a case of RDD with bone marrow involvement in a 5-year-old child. Through this case report, we aim to emphasize the importance of bone marrow exploration in patients with RDD and presenting cytopenias.

## 2. Case Presentation

A 5-year-old female from nonconsanguineous marriage, with a medical history of snoring without sleep apnea, had presented asthenia, gingival bleeding, and diffuse bone pain evolving for three months, without associated fever. Before admission to our hospital, the patient had visited a general practitioner who ordered blood tests. Initial cell blood count showed bicytopenia (microcytic hypochromic anemia at 10.1 g/dL and thrombocytopenia at 100 G/L) with low reticulocytes at 70250/mm^3^. The patient was referred to the hospital for further investigations. Physical examination showed pallor, gingival hypertrophy, and petechiae over the abdomen. Palpation revealed painless and bilateral cervical lymphadenopathy, without splenomegaly or hepatomegaly. The skeletal examination showed several bony nodules on the forehead, mandible, wrists, and knees.

Cell blood count revealed microcytic hypochromic anemia (hemoglobin = 10.4 g/dL; MCV = 77.4 fL; MCHC = 26.1 pg), thrombocytopenia (platelet count = 84 G/L), and normal white blood cell count at 4.56 G/L (neutrophils = 3.11 G/L; lymphocytes = 1.2 G/L). The erythrocyte sedimentation rate was elevated at 57 mm/hour. Biochemical parameters objectified hyperferritinemia at 241 ng/mL and a high level of lactate dehydrogenase at 688 U/L.

**Figure 1:**
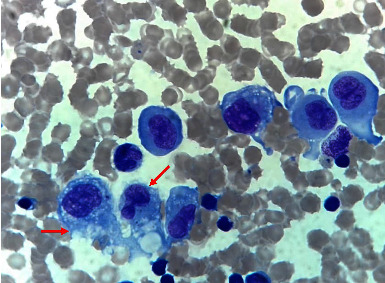
Bone marrow aspiration at high magnification ×100 showing bone marrow infiltration by histiocytic cells. Large histiocytes with hypochromatic nuclei and abundant vacuolated cytoplasm (arrow).

**Figure 2:**
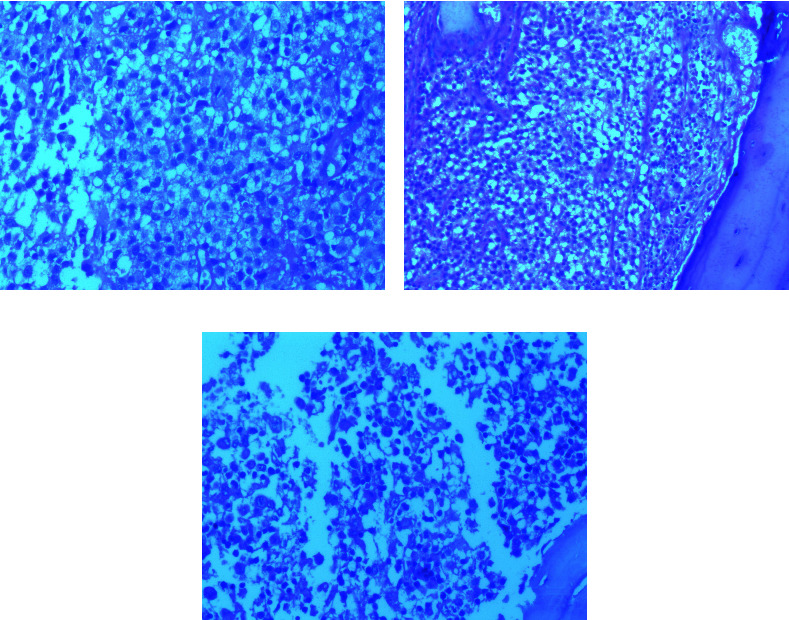
Biopsies showing infiltration by histiocytic cells. (a) Cervical lymph node biopsy, hematoxylin, and eosin, ×20. (b) Bone biopsy, hematoxylin, and eosin, ×10. (c) Bone marrow biopsy, hematoxylin, and eosin, ×10.

**Figure 3:**
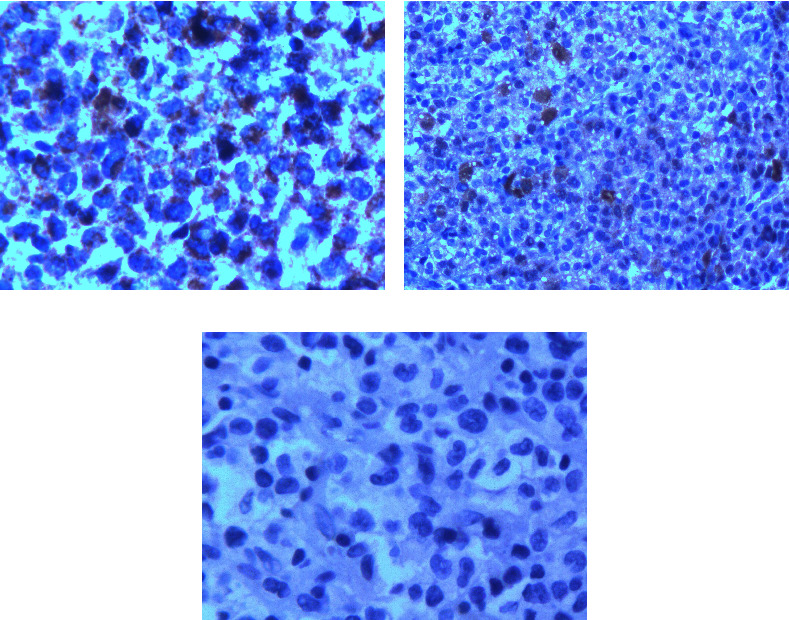
Immunohistochemical staining of lymph node biopsy. (a) CD68 expression by histiocytic cells. (b) S100 protein expression by histiocytic cells. (c) Histiocytic cells lack CD1a expression.

Peripheral blood smear showed anisopoikilocytosis with mild hypochromia, without platelet clumping. Bone marrow aspiration was normocellular with the absence of megakaryocytes. It was infiltrated by 31% of large histiocytes with hypochromatic nuclei and abundant vacuolated cytoplasm, without emperipolesis (Figure 1). An infiltration by mature histiocytic cells was also observed in bone marrow, lymph nodes, and bone biopsies (Figure 2). Immunohistochemically, the histiocytes were positive for CD68 and S100 protein but negative for CD1a (Figure 3). A diagnosis of RDD involving bone marrow and bones was made. No concurrent malignancy was detected. Lymph node tuberculosis was ruled out because there was no histological evidence. Antinuclear antibodies, anti-DNA antibodies, and rheumatoid factor were absent, which ruled out an associated autoimmune disease. Serological tests for hepatitis B and C, syphilis, and cytomegalovirus were negative.

Neck ultrasound confirmed the presence of bilateral cervical lymphadenopathies. Chest and neck CT scan revealed bilateral lytic bone lesion involving both scapulas and bilateral cervical lymphadenopathies.

The patient has first received analgesics as a symptomatic treatment. On day 6 of admission, the patient's clinical course was deteriorated. She presented signs of septic shock: fever at 40°, hypotension with increased C reactive protein at 47 mg/L. The patient was transferred to the pediatric intensive care unit for appropriate management. Empirical antibiotic therapy was initiated (imipenem + vancomycin). However, the blood culture was negative without any microbiological evidence. The patient died one week after admission to the hospital before the final establishment of RDD diagnosis.

## 3. Discussion

Histiocytosis is a heterogeneous group of rare diseases distinguished by the accumulation of cells derived from macrophages or dendritic cells in diverse tissues and organs of children and adults [5, 8]. RDD is a rare and benign histiocytic disorder first described in 1965 by Destombes [1]. In 1969, Rosai and Dorfman described the same entity as “sinus histiocytosis with massive lymphadenopathy” [2]. In 1987, the Histiocyte Society classified RDD as a nonLangerhans cell histiocytosis [3]. Recently, according to the 2016 revised histiocytosis classification, RDD belongs to the “R Group” of histiocytosis which includes familial and sporadic RDD, while cutaneous RDD belongs to the “C Group” [5]. Sporadic RDD includes the classical nodal RDD, extranodal RDD, neoplasia-associated RDD, and immune disease-associated RDD [5]. RDD commonly affects children and young adults (mean age: 20.6 years) and frequently males of African descent [4–6, 9]. In our case, the patient was a 5-year-old female child, whereas the patients were male adults in Huang et al. and Zanelli et al. case reports [7, 10]. The pathophysiology of RDD is poorly understood [4]. It was considered as a reactive and non-neoplastic histiocytic disorder; however, recent studies described NRAS, KRAS, ARAF, and MAP2K1 mutations in nodal and extranodal RDD [4, 6]. Studies have supposed that herpes viruses, cytomegalovirus, Epstein-Barr virus, and HIV may be potential inciting agents, but no causative link has been demonstrated [4, 6]. In our patient, cytomegalovirus serology was negative. The other viral serologies were not performed.

Mutations in the SLC29A3 gene have been reported in familial cases of RDD [4, 6]. Sequencing studies to identify a germline predisposition to RDD were not pursued in our patient.

In classical sporadic RDD, the most frequent clinical presentation is bilateral painless massive lymphadenopathy with associated fever, night sweats, asthenia, and loss of weight [5]. Mediastinal and inguinal nodes may be associated [5, 9]. Extranodal manifestations are present in about 40% of cases, and the most frequent sites involved are skin, nasal cavity, bone, soft tissue, orbital tissue, and central nervous system [4, 5, 9]. The medical history of snoring in our patient suggests the possibility of Rosai–Dorfman disease with associated nasal cavity localisation. However, a CT scan of the nasal cavity and paranasal sinuses was not performed.

RDD diagnosis is confirmed by histology [5, 9]. Lymph node biopsy is characterized by the accumulation of large histiocytic cells with hypochromatic nuclei, abundant and pale cytoplasm, with emperipolesis which is a characteristic morphological feature of RDD [5, 8, 9]. However, emperipolesis is not specific and not required for RDD diagnosis [4]. In our patient, there was no observed emperipolesis. Biopsies of extranodal sites show the same histological features of histiocytes [9]. Immunohistochemically, the proliferative histiocytes are S100, CD68, and CD163 positive and are CD1a and CD207 negative [4, 5].

Bone involvement is reported in 5% to 10% of RDD cases [6]. Its common manifestation is bone pain with osteolytic bone lesions [5, 6].

Hematologic abnormalities in RDD include normocytic normochromic anemia (67% of cases), leukocytosis with neutrophilia (60% of cases), and thrombocytopenia [6]. As in Huang et al. case, our patient presented with anemia and thrombocytopenia, while in Zanelli et al. case, the patient had a mild pancytopenia [7, 10]. Bone marrow infiltration is rarely reported in the literature [6, 7, 10]. The first extranodal RDD involving the bone marrow was reported by Huang et al. in 2006 [7]. Bone marrow exploration was not performed in the past as a staging procedure, which explains the rare reported cases of bone marrow involvement in RDD [7, 10]. According to the consensus recommendations for the diagnosis and management of RDD by the American Society of Hematology, all patients with unexplained cytopenias or abnormal peripheral blood cells require bone marrow aspiration and biopsy [6].

Abnormal laboratory test results such as hyperferritinemia and high erythrocyte sedimentation rate are not specific and may be related to inflammation or infection [5]. It is recommended to carry out antinuclear antibodies and rheumatoid factor tests in order to screen for an associated immune disease [6]. These tests were negative in our patient, thus, there was no evidence of associated autoimmunity.

In our case, there was no malignancy detected, whereas Zanelli et al. reported a case of RDD involving bone marrow associated with acute myeloid leukemia [10].

RDD patients need a long-term follow-up searching for other possible tissues infiltration [11].

Many therapeutic strategies exist and depend on RDD manifestations, among these treatments: corticosteroids, chemotherapy, radiotherapy, immunomodulatory therapy, and surgery [6, 9].

Sporadic RDD has generally a good prognosis, especially nodal disease which is usually self-limited [4, 6]. Bony RDD prognosis is favorable in general [6]. Multifocal and extranodal RDD, including bone marrow involvement, have a poor prognosis [6, 7, 10].

## 4. Conclusion

RDD is a rare and benign histiocytic disorder. However, bone marrow involvement in RDD is associated with unfavorable prognosis. Hematologists and pathologists must be aware of the possible infiltration of bone marrow despite its rarity. According to the American Society of Hematology consensus recommendations for the diagnosis and management of RDD in 2018, it is required for RDD patients with unexplained cytopenias or abnormal peripheral blood cells to undergo bone marrow aspiration and biopsy.

## Data Availability

The data used to support the findings of this study are included within the article.
